# Last Generation Triazoles for Imported Eumycetoma in Eleven Consecutive Adults

**DOI:** 10.1371/journal.pntd.0003232

**Published:** 2014-10-09

**Authors:** Yoann Crabol, Sylvain Poiree, Marie-Elisabeth Bougnoux, Christophe Maunoury, Stéphane Barete, Valérie Zeller, Cédric Arvieux, Samuel Pineau, Karima Amazzough, Marc Lecuit, Fanny Lanternier, Olivier Lortholary, the French Mycosis Study Group

**Affiliations:** 1 Centre d’Infectiologie Necker Pasteur, Université Paris Descartes, Sorbonne Paris Cité, Institut Imagine, Hôpital Universitaire Necker-Enfants malades, APHP, Paris, France; 2 Service d’Imagerie Médicale, Hôpital Necker-Enfants malades, Paris, France; 3 Laboratoire de parasito-mycologie, Hôpital Universitaire Necker-Enfants malades, CNRS URA3012, Paris, France; 4 Unité de Médecine Nucléaire et TEP, Université Paris Descartes, Hôpital Européen Georges Pompidou, Paris, France; 5 Service de Dermatologie, Hôpital Tenon, APHP, Université Pierre et Marie Curie, Paris, France; 6 Service de Médecine Interne, Groupe Hospitalier Diaconesses Croix Saint-Simon, Paris, France; 7 Service de maladies infectieuses et réanimation médicale du Centre hospitalier universitaire de Rennes, Rennes, France; 8 Hôpital Universitaire de Nantes, Service des Maladies Infectieuses et Tropicales, Nantes, France; 9 Unité de Biologie des Infections, Institut Pasteur, Inserm U1117, Paris, France; 10 Unité de Mycologie Moléculaire, Institut Pasteur, Centre National de Référence Mycoses Invasives et Antifongiques, Paris, France; Fundação Oswaldo Cruz, Brazil

## Abstract

**Background:**

Optimal management of eumycetoma, a severely debilitating chronic progressive fungal infection of skin, disseminating to bone and viscera, remains challenging. Especially, optimal antifungal treatment and duration are ill defined.

**Methodology/Principal Findings:**

We conducted a monocentric retrospective study of 11 imported cases of eumycetoma treated by voriconazole or posaconazole for at least 6 months. Response to treatment was assessed through evolution of clinical and magnetic resonance imaging (MRI). (1→3) ß-D-glucan (BG) and positron emission tomography using [18F] fluorodeoxyglucose (PET/CT) results were also assessed. Identified species were *Fusarium solani complex* (n = 3); *Madurella mycetomatis*, (n = 3), and *Exophiala jeanselmei*, (n = 1). Moreover, two coelomycetes and one phaeohyphomycetes strains without species identification were retrieved. Serum BG and PET/CT were abnormal in 7/8 and 6/6 patients tested, respectively. Patients received last generation azoles for a mean duration of 25.9±18 months. Complete response (major clinical and MRI improvement) was observed in 5/11 patients, partial response (minor MRI improvement or stable MRI findings) in 5 and failure (MRI evidence of disease progression) in one, with a 73±39 [6–132] months mean follow-up. Relapse occurred in 2 patients after treatment discontinuation. Optimal outcome was associated with fungal species, initiation of last generation triazole therapy (<65 months since first symptoms), negative serum BG and PET/CT normalization.

**Conclusions/Significance:**

MRI, PET/CT and serum BG appear as promising tools to assess optimal time of antifungal treatment for eumycetoma.

## Author Summary

Eumycetoma is a severe chronic progressive fungal infection of skin and ultimately bone or viscera that affect mainly people with low economic status. Optimal treatment of this condition relies on medical and often surgical therapy and remains challenging because of lack of gold standard therapy and high rate of relapse after treatment discontinuation. In this retrospective study we assessed whether modern triazoles (voriconazole and posaconazole) suited to eumycetoma treatment and if tailored treatment duration, based on serial evaluation of a serum biomarker of fungal infection (Serum (1→3) ß-D-Glucan, (BD)), magnetic resonance imaging (MRI) or positron emission tomography using [18F] fluorodeoxyglucose (PET/CT) would be useful. We found that modern triazoles were efficient treatments of eumycetoma, allowing complete or partial response in 10/11 of patients, without significant side effects. Moreover, patients with treatment discontinuation based on normalization of BD, MRI or PET/CT seemed to have better long-term outcome than those with clinical cure but still abnormal BD or imaging results.

## Introduction

First described in 1642 by Kaempfer in his dissertation in the University of Leiden and then by John Gill as “Madura Foot” in 1842, mycetoma is a chronic progressive and pseudotumoral granulomatous infection of skin, subcutaneous tissues and ultimately bone or viscera caused by fungi (eumycetoma) or bacteria (actinomycetoma) [1]. Young male adults of low socioeconomic status particularly manual workers in poor resource areas are the worst affected. Eumycetoma prevails in the belt that stretches between the 15th South and 30th North parallels, especially in Sudan and India, where drought could favors fungal growth in plant material including acacia and cow dung. Rural barefoot activities favor fungus transmission to human through subcutaneous contaminated thorn pick, which then spread locally or through the lymphatic system, and rarely through the bloodstream [2], [3].

The eumycetoma clinical triad consists in painless subcutaneous mass, sinus formation and sero-purulent discharge that contains grains, aggregates of the fungal hyphae [4]. Among black grained-mycetoma, always of fungal origin, *Madurella mycetomatis* is the most prevalent causative agent [5]. Among white grained-mycetoma, *Scedosporium boydii, Acremonium falciforme and Fusarium spp* have been most frequently reported [5].

Eumycetoma is not self-healing and spontaneously leads to severely debilitating limb lesions with severe socioeconomic consequences [6], making it currently one of the neglected tropical disease according to WHO [7].

Optimal management of eumycetoma remains ill defined and mostly relies on surgery and prolonged oral first generation antifungal treatment, when available. Indeed, surgery alone is associated with a 10–25% relapse rate [8]. In contrast, since 1984, itraconazole, ketoconazole and terbinafine have been evaluated in limited open prospective trials (*i.e.* maximal total number of patients recruited being 20), in association or not with surgery, and disclosed a 70–80 % response rate, with an acceptable tolerance after up to 24 months of therapy and less than 10% relapse [9]–[13]. More recently, preliminary reports mainly from non-endemic countries using posaconazole and voriconazole have demonstrated *in vitro* activity and suggested encouraging *in vivo* efficacy [14]–[22].

In order to better assess last generation triazoles efficacy and their minimal treatment duration, we conducted a multicentric retrospective study from 2002 to 2013 of 11 adult patients with eumycetoma treated by voriconazole or posaconazole for at least 6 months with a follow-up of at least 6 months at the Centre d’Infectiologie Necker-Pasteur, Paris, France.

## Methods

### Ethics statement

This study was conduced in compliance with the Institutional Review Board Paris Necker. In accordance with French law regarding retrospective studies, oral consent was obtained from each patient.

### Patients

Through the French National Reference Center for Invasive Mycoses and Antifungals (NRCMA, Institut Pasteur, Paris), we retrospectively collected all adult cases of proven eumycetoma treated with voriconazole or posaconazole for a minimum period of 6 months and evaluated each of them at least once at the Centre d’Infectiologie Necker-Pasteur from January 2002 to December 2013.

All patients underwent magnetic resonance imaging (MRI) and/or positron emission tomography using [18F] fluorodeoxyglucose (PET/CT) before recent triazoles start and then every 6 to 12 months according to physician opinion and patient evolution. Moreover, ten CT scanner were performed among 4 patients. Six chest and abdominal CT scanner were performed in the patient 5 with lung involvement.

A standardized form was used to collect information regarding age, sex, origin, place of contamination, medical background, immunodepression, date and localization of first symptoms, date and modalities of microbiological and/or pathological diagnosis, prior antifungal treatment and surgery, current triazole therapy, dosage regimen, route of administration, trough serum levels and side effects potentially attributable to one of the two tested triazoles.

### Mycological identification

Excised grains were incubated at 30°C on liquid blood agar medium and Sabouraud glucose adding with chloramphenicol during at least 7 days. All isolates were identified by phenotypic methods (macroscopic and microscopic aspect on Sabouraud, PDA and Malt extract media, growth at 37°C, determination of conidiogenesis by using slide culture on Malt extract agar for *Exophial*a sp. and *Fusarium* sp. isolates), and by sequencing of the ITS and D1/D2 regions of the gene coding ribosomal RNA by using universal primers (V9D [23]/LS266 [24] and NL1/NL4 [25] primers respectively).

### (1→3) ß -D-Glucan detection in serum samples

Serum (1→3) ß -D-Glucan (BG) levels were determined with the Fungitell test kit (Associates of Cape Cod, Inc.,Cape Cod, MA), according to the manufacturer's instructions. The results of a kinetic colorimetric assay performed at 37°C were read at 405 nm for 40 min. The BG concentrations in samples were calculated automatically by using a calibration curve established with standard solutions ranging from 6.25 to 100 pg/ml. This assay is reported continuously for results between 31–500 pg/mL, and as>500 pg/mL for values above this range. BG levels higher than 80 pg/ml were considered to be positive, as defined by the manufacturer. Serum assays were performed in duplicate

### Trough concentration of posaconazole and voriconazole determination

A drug assay was performed for 26 samples among 8 patients using a previously published high-performance chromatography-UV detection method [26].

### Definitions and evaluation criteria

Immunodepression included history of diabetes, cancer, chronic renal disease, HIV infection, autoimmune disease or immunosuppressive therapy. Search for immune deficiency included anti-HIV antibodies, T, B and NK lymphocytes phenotyping and protein electrophoresis. Organ involvements were defined by abnormal MRI or surgical appearance compatible with eumycetoma. Osteitis was defined by T1 weighted hypointense and T2 weighted hyperintense signal of bone.

Clinical response to treatment was assessed by a clinical score based on the presence of pain, inflammation signs and spontaneous drainage. Clinical responses were classified as “major” defined by a score equal to zero or “partial” defined by a decrease in clinical score. Stable clinical response and clinical failures were also considered. Biological response was assessed through BG serum levels. Major, minor, stable and worsened BG responses were defined by normalization, decreasing, unchanged or increasing values, respectively. MRI response, based on the Mycetoma Skin, Muscle, Bone Grading already reported [27], was assessed by a single expert radiologist (SP) through comparison of site, size and contrast enhancement of main lesions. MRI response was notified as major, minor, stable or failure in case of pathologic hyper T2 signal complete disappearance, improvement by at least 50%, stability or in case of new lesions occurrence, respectively. The presence of a “Dot in the circle” pattern, *i.e.* conglomerate areas of small round discrete T2 weighted hyperintense lesions surrounded by a low-signal-intensity rim with central dot, highly suggestive of mycetoma [27], [28] was also analyzed. PET/CT response relied on maximum Standard Uptake Value (SUV) comparison in a single nuclear medicine department (CM). Major, minor, stable responses and failure were defined by negativation, improvement (more than 30% decrease), no change (less than 30% change) or worsening (more than 30% increase) of max SUV values, respectively.

End of treatment (EOT) time was defined as time of treatment discontinuation or last available evaluation. EOT response was defined as “complete response (CR)” in case of clinical score negativation, and major MRI response, as “failure” if one of these parameters remained unchanged or had deteriorated or as “partial response (PR)” otherwise.

Risk for underdosing was defined by low posaconazole or voriconazole trough concentration (<1 µg/mL) or obvious non-adherence reported by the physician in charge of patient.

### Statistical analysis

Continuous data were described with descriptive statistics, including mean±SD and/or median [range] as appropriate and categorical data with frequencies (%). Categorical data were analyzed by univariate analysis with Fisher's exact test as appropriate and continuous data by nonparametric Mann-Whitney test. Univariate analysis was used to identify factors associated with overall complete response at end of treatment (EOT). P ≤.05 was considered statistically significant. Antifungal treatments which duration was shorter than six months were excluded from statistical analysis. Statistical analyses involved use of SPSS software.

## Results

### Patients

Eleven cases of proven eumycetoma were identified during the study period (Table 1). Median age at the time of first symptoms was 28.8 [10.0–56.3] years. All patients were from African descent (Senegal, n = 3, Mali, n = 2, Brazil, Martinique, Tchad, Mauritania, Togo, and Mayotte, n = 1 each), native from Western or Central Africa (8/11). All but one patient had been likely contaminated in Western or Central Africa, and 4/11 reported a preceding trauma with thorn or stone. Initial sites of lesion were mainly foot or ankle (9/11). Secondary skin and soft tissue infection was reported in 3/11 patients. Because of debilitating progression of the disease, 5/11 patients had to leave their job.

**Table 1:** Patients and eumycetoma characteristics at diagnosis.

Patient Number		1	2	3	4	5	6	7	8	9	10	11
**Origin**		Mali	Martinique	Senegal	Senegal	Tchad	Mali	Senegal	Brazil	Mauritania	Mayotte	Togo
**Sex/Age at 1st symptoms, year**		M/26	M/41	M/29	M/31	M/19	M/35	M/49	M/18	M/10	M/18	M/56
**Place of contamination**		Mali	Martinique	Senegal	Senegal	Tchad	Mali	Senegal	Angola	Senegal	NA	Togo
**Initial Site**		Malleolar Left Foot	Malleolar Right Foot	Top of Right Foot	Top of Right Foot	Right lumbar Back	Right Knee	Internal Right Foot	Top Left Foot	Top Left Foot	Top Left Foot	Right ankle
**Pre New azole:**												
	**Organ Involvement**	SB	SMBJ	SMBJ	SB	SBMNV	SB	S	SM	SB	SMBJ	SBJ
	**Details**	Foot bone, tibia	Foot bone, tibia	Foot bones	Foot bones	Diaphragm, right lung, kidney and psoas	Femur			Foot bones	Foot bones	Foot bone, tibia,fibula
	**Max SUV (PET/CT)**	4,2	NP	15,2	NP	15	NP	NP	NP	4,9	6,6	1 focus
	**BD glucan (pg/ml)**	443	305	500	NP	254	80	NP	NP	NP	NP	NP
**Mycetoma sp**		Phaeohyphomycete	*Exophiala jeanselmei*	Phaeohyphomycete	*Fusarium solani*	*Madurella mycetomatis*	*Madurella mycetomatis*	Coelomycete	*Fusarium solani*	*Madurella mycetomatis*	*Fusarium solani*	Coelomycete
**Grain Color**		Black	Black	Black	White	Black	Black	Black	White	Black	White	Black
**Time to Diagnosis, months**		120	268	50	156	36	7	58	318	36	60	7
**Superinfection**		No	Erysipelas	No	No	Lung Abscess	No	No	No	Erysipelas	No	No
**Loss of job**		No	No	No	Yes	Yes	Yes	NA	NA	Yes	Yes	Yes

B: Bone; J: joint; M: Male; M: muscle; N: node; NA: Not available; NP: Not Performed; S: Skin; SF: Secondary Failure; V: visceral; VCZ: voriconazole.

At diagnosis, most of them (6/11) had cardiovascular risk factor, mainly hypertension. Two patients had chronic HBV infection and 4 patients type II diabetes or chronic kidney disease. HIV serology was negative, gammaglobulin serum levels and T, B, NK lymphocytes counts were normal in all patients. Consanguinity was present in 3/11 patients.

### Diagnosis

Diagnosis was established in France in all but one patient with a median time of 58 [7–318] months since first symptoms. Grains, mostly of black color (8/11) were seen in every histopathological examination thereby confirming mycetoma. Microscopic examination and mycological culture were positive in 7/11 and 10/11 cases, respectively. Identification was possible to the species level in 7/10 cases, through exclusive phenotypic methods in 4 cases (*Fusarium solani* complex, n = 2; *Madurella mycetomatis*, n = 2) and ITS 1/2 sequencing in 3 cases (*Madurella mycetomatis*, n = 1; *Fusarium solani complex*, n = 1; *Exophiala jeanselmei*, n = 1). In 4 cases, identification was only possible to the class level of Coelomycetes (patient 7 and 11), and Phaeohyphomycetes (patient 1 and 3). Coelomycetes class identification relied on ITS 1/2 sequencing. Phaeohyphomycete class identification relied on the presence of pigmented molds on microscopic histopathological examination, with negative cultures (patient 3) and phenotype analysis of colonies with inconclusive ITS 1/2 sequencing results (patient 1).

### Prior treatments

Before last generation azole therapy, 5 patients had already received antifungal treatments such as ketoconazole (n = 4), itraconazole (n = 2), fluconazole or terbinafine (n = 1, each) (see Table 2). Primary clinical failure had occurred with fluconazole, or with shorter than 3 months treatment regimens. Eight patients had already undergone a surgical lesion resection that induced median clinical remission duration of 162 [19–288] months before relapse.

**Table 2:** Treatment and outcome characteristics of eleven patients with eumycetoma.

Patient Number		1	2	3	4	5	6	7	8	9	10	11
**Prior antifungal treatment name duration, months and outcome**		KTZ 0.5 PF	None	KTZ 72 SF FCZ 8 PF KTZ 22 R	TBF 3 NA ITZ 6 NA	ITZ 6 R	KTZ 0.3 R	None	None	None	None	None
**New Azole antifungal treatment**												
	**Name**	VCZ	PCZ	VCZ	VCZ/PCZ	VCZ/PCZ	PCZ	VCZ	VCZ	PCZ	VCZ	VCZ
	**Duration, months**	9	70	9	24	40	22,5	30	35	18,5	18	9
	**Combination therapy**	No	No	No	No	TBF	TBF	No	No	FC	TBF	No
	**Underdosage risk factor**	Yes	No	No	Yes	Yes	Yes	No	Yes	No	No	No
**MIC VCZ/PCZ**		**NA/NA**	0.25/0.125	NA/NA	NA/NA	NA/NA	NA/NA	0.014/0.014	4/8	NA/NA	8/NA	8/8
**Pre/per new azole surgery number**		1/0	1/0	4/0	0/0	3/2	1/2	0/0	1/1	3/2	1/0	0/1
**Time to:**												
	**Surgery, months**	120	12	50		0	7		144	120	60	7
	**Modern azole treatment, months**	414	267	515	204	119	65	63	330	290	114	9
**EOT response**		**PR**	**CR**	**PR**	**PR**	**Failure**	**CR**	**CR**	**PR**	**CR**	**PR**	**CR**
	**EOT clinical response**	Major	Major	Major	Minor	Stable	Major	Major	Major	Major	Minor	Major
	**EOT MRI response**	Stable	Major	Minor	NP	Worse	Major	Major	Minor	Major	Minor	NP
	**EOT PET/CT response**	Minor	Major	Minor	NP	Worse	Major	Major	Minor	Major	NP	NP
	**EOT BD Glucan response, (value)**	Stable (472)	Major (<80)	Stable (480)	237 (post relapse)	Worse (>500)	Major(<80)	NP	? (123)*	NP	390 (post relapse)	NP
	**Follow up, months since new azole discontinuation**	Ongoing	10	Ongoing	132	Ongoing	Ongoing	72	Ongoing	72	68	83
**Relapse after new azole discontinuation, months**			No		Yes, 8			No		No	Yes, 11	No

B: Bone involvement; CPK: creatinine phosphokinase; CR: Complete Response; D: drainage; I: inflammation; ITZ: itraconazole; J: Joint involvement; KTZ: ketoconazole; M: Muscle involvement; Node involvement; P: pain; PCZ: posaconazole; PF: Primary failure; PR: Partial Response; R: relapse; S: soft tissue involvement; SUV: Standard Uptake Value; V: Visceral involvement; VCZ: voriconazole.

* Patient 8 only had one post last generation triazole treatment dosage of BG so that evolution couldn’t be assessed.

### Evaluation at the time of voriconazole or posaconazole initiation

**Figure 1:**
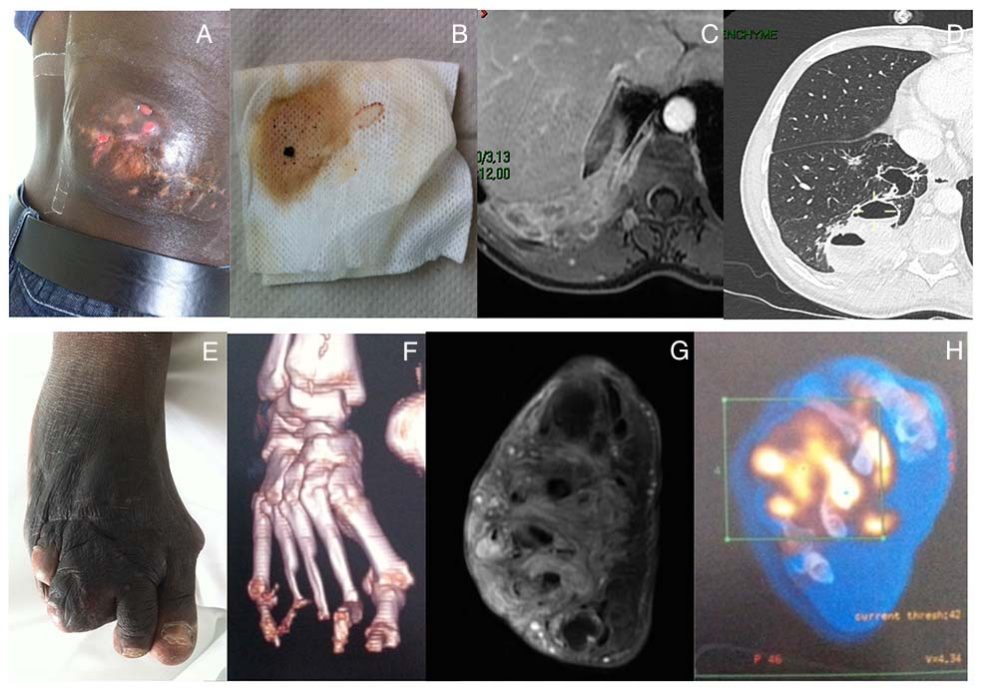
Organ involvement in patients 4 and 5. Lumbar tumefaction with draining sinuses (A). Black grains drainage (B). Paraspinal abdominal eumycetoma extension on abdomen MRI LAVA sequence (C) and lung extension on lung CT scanner (D) in patient 5. Clinical (E) second and third metatarsal destruction on 3D reconstruction CT (F) of foot. Foot mycetoma aspect on Axial T1 Fat Sat Gadolinium MRI (G) and PET/CT (H) in patient 4.

At the time of initiation of last generation triazoles, all patients were symptomatic and had pain due to osteitis, local inflammation or purulent discharge (see Table 2 and Figure 1). MRI was abnormal in all 11 cases, showing soft tissue, osteolytic bone (mainly talus, calcaneus and metatarsal), muscle, articular and visceral lesions (lung, diaphragm and kidney) in 11, 8, 5, 4 and 1/11 cases respectively. A “dot in the circle” pattern was noticed in soft tissue in 7/11 cases (picture 3A). Serum BG was tested in 8/11 cases and was positive in 7/8 cases (median value 305 [80–500] pg/ml; normal<80 pg/ml)). There was no correlation between BG value, lesion size evaluated by MRI or CT scanner nor SUV max. All 6 patients studied had abnormal PET/CT with a median SUV max of 6.6 [4.9–15.2].

### Last generation triazole and surgical treatment

Last generation triazoles were initiated as primary therapy (n = 8) or as secondary therapy following failure from prior treatment (n = 3) (see Table 2). 6/11, 3/11 and 2/11 patients were treated with voriconazole (200 to 350 mg BID outside meals), posaconazole (400 mg BID with food) or switch from posaconazole to voriconazole for failure, respectively. Three patients were treated with combination antifungal therapy including terbinafine (n = 2) or flucytosine (n = 1). Additional small eumycetoma surgical excision was performed in 5/11 patients. At last evaluation, after a mean and median uninterrupted duration of 22.2±18.3 and 18 months respectively, treatment had been discontinued for completion or toxicity in 5/11 and 1/11 patients, respectively, or was ongoing in 5 cases.

### Response to treatment and side effects

**Figure 2:**
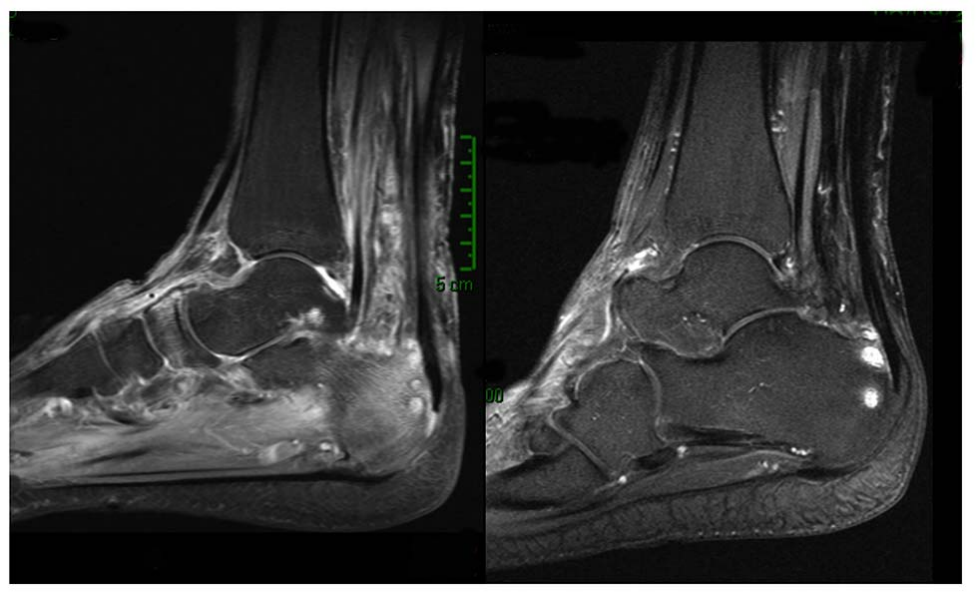
Major MRI Response in patient 2. Major MRI response in Patient 2 with disappearance of periachillean tissular infiltration and of talus bone edema. Slight talus contrast enhancement persistance on T1 Gado Fat Sat sequence.

**Figure 3:**
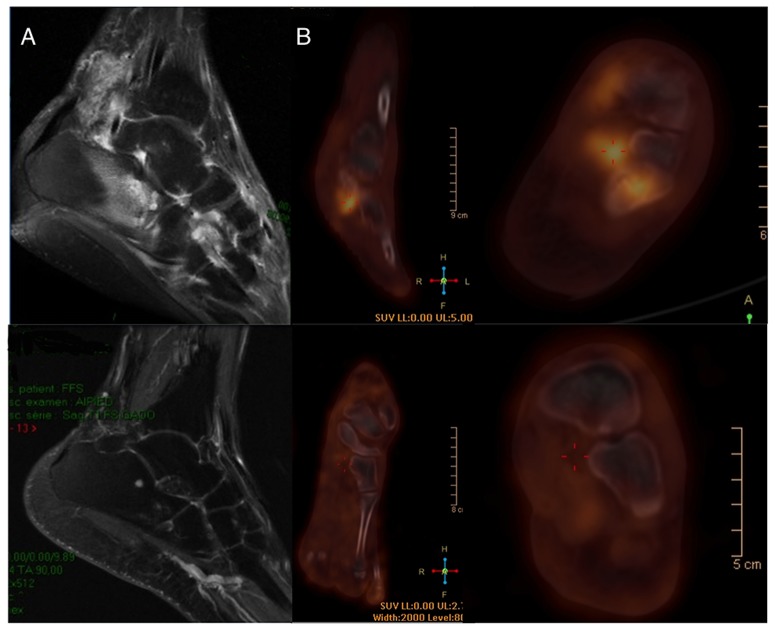
Major MRI and PET/CT responses in Patient 9. Left foot T1 Fat Sat Gadolinium enhanced MRI (A): Disappearance of periachillean tissular infiltration with the dot in the circle pattern, talus bone edema disappearance and slight talus contrast enhancement persistence. Disappearance of talus and tarsal hypermetabolism on left foot PET/CT (B).

EOT response was complete, partial and null in 5/11 (45.4%), 5/11 (45.4%) and 1/11 (9.1%) patients, respectively (see Table 2 and Figure 2 and 3). Among partial responders, 3/5 had a negative clinical score and 2/5 still complained of pain. All 3 patients with negative clinical score had minor MRI improvement or stable MRI findings. The patient with failure had stable clinical score but MRI evidence of progression of mycetoma to lung and paraspinal region. Complete responders (CR) received at least 9 months of uninterrupted last generation azole treatment. Six patients whose triazoles treatment was discontinued had a mean follow up of 73±39 [6–132] months. Among them, relapse occurred in 2 patients, 8 and 11 months after treatment discontinuation, respectively.

EOT major clinical, MRI, PET/CT and BG responses were achieved in 8/11, 4/9, 4/8 and 2/6 cases, respectively after a mean duration of triazole therapy of 25.9±18.0 months. In all patients, slight MRI contrast enhancement persisted in soft tissue at last evaluation.

Side effects were reported in 3/11 patients treated with voriconazole and included chronic cholestatic hepatitis, transitory visual disturbance (related to high voriconazole trough of 5.1 µg/mL) and muscle pain with CPK elevation. Of note, none of the patients presented skin lesion, pain suggestive of fluorosis or significant drug-to-drug interaction.

### Comparison of patients with complete and partial or failure responses

CR slightly differed from non-CR regarding patient's history, eumycetoma disease or treatment related characteristics. CR patients tended to be older at first symptoms (median age of 40.6 years [10–56.3] and 22.5 years [18]–[31], respectively, p = 0.13) and more often naive of antifungal treatment (5/5 (100%) and 3/6 (50%) patients, respectively, p = 0.38). CR presented a trend towards earlier diagnosis of eumycetoma (median time to diagnosis 36 [7–268] and 90.0 [36–318] months respectively, p = 0.18), earlier last generation azole treatment (median time to new azole treatment of 65 [9–290] and 267 [114–515] months respectively, p = 0.13) and more combination therapies (2/5 (40%) and 1/6 (16.7%) patients respectively, p = 0.38). None of CR patients was infected with *Fusarium solani* or unidentified black fungi, whereas 5/6 (83.3%) of non-CR were infected with one of those species or unidentified black fungi (p<0.05). Frequent lack of fructification of collected strains explained difficulties to obtain readable MIC values for all patients. MIC values were available in 5 patients, 3 with CR and 2 with PR. Patients with CR tend to have been infected with a fungus exhibiting lower triazole MIC values than those found in patients with PR or failure (median and range MIC 0.125 µg/L [0.014–8] and 6 µg/L [4]–[8], respectively, p =  0.4)

Remembering the small population size described here, patients only treated with posaconazole had nevertheless a higher rate of complete response than that found in other patients (3/3 (100%) vs 2/8 (25%) respectively, p = 0.06). CR received longer duration of uninterrupted last generation azole treatment (30±23.6 and 15.7±10.6 months, respectively, p = 0.18). CR also had less azole under dosage risk factors (1/5 (20%) and 4/6 (66.7%) respectively, p = 0.24). EOT BG and PET/CT major responses were associated with complete response at EOT (p = 0.06 and p<0.05 respectively). All of the two relapses occurred in the 2 non-CR patients and consisted in local inflammatory signs reappearance occurring after a mean duration of triazoles therapy of 21±4.2 months.

## Discussion

In this retrospective monocentric study, we describe detailed clinical, biological, MRI and PET/CT responses of eleven patients with eumycetoma treated by a last generation triazole in Paris. Despite obvious limitations due to the design and small sample size, it is to the best of our knowledge the largest reported case series of eumycetoma treated with last generation antifungal triazoles and the first to assess the contribution of fungal biomarkers and MRI PET/CT in eumycetoma evaluation.

Complete or partial response was observed in 10/11 patients treated with last generation triazoles with or without additional surgery. This result is in agreement with previously limited published data showing 83% (5/6) complete or partial response rate reported with last generation triazoles in the literature [17]. Interestingly, the only case qualified as non-responder (patient 5) was the one with the most severe visceral involvement due to *Madurella mycetomatis*. Success of modern triazole despite failure of previous azole treatments might be explained by the fact that voriconazole has been shown in vitro to be less susceptible to melanin binding than other antifungals and therefore more bioavailable [29].

 In previous studies, longer treatment duration and absence of history of disease recurrence were reported as significant predictors of increased odds of cure from eumycetoma [30]. Here, those with complete response were also more often naive of azole treatment and had a trends toward a longer median treatment duration, of at least 9 months. Moreover, our results emphasize the need to shorten time to diagnosis and deliver prolonged and uninterrupted treatment with optimal observance and therapeutic drug monitoring (TDM). TDM has already been shown to reduce drug discontinuation due to adverse events and improve treatment response in invasive fungal infections [31], [32]. Finally, PR or failure tended to be associated with *Fusarium solani* complex sp, higher MIC of triazole and voriconazole vs. posaconazole use. Whether these findings reflect higher virulence of certain fungal species and/or suboptimal pharmacokinetic-pharmacodynamic end points achievement remains unclear. However, posaconazole is known to exhibit lower MIC values than voriconazole against *Fusarium* sp [33], *M. mycetomatis* [20] and *E. janselmei* [34] which might partly explains these results.

Both early diagnosis of mycetoma and recognition of its fungal or bacterial origin are critical [6]. While subtle differences in inflammation, speed pace, grain color or radiologic pattern of bone lesions have already been reported, early distinction between eumycetoma and actinomycetoma remains challenging [2], [27]. Here, the “dot in the circle” pattern was present in 7/11 patients and was already reported as an early sensitive and very specific MRI pattern of eumycetoma and actinomycetoma [27], [28]. BG is a cell wall component of most fungal species. It has been reported as an interesting tool for early diagnosis of invasive fungal infections in patients with haematological malignancies [35] and *Pneumocystis jirovecii* pneumonia [36]. Importantly, serum BG was positive in 7/8 studied, suggesting its potential diagnostic role to discriminate eumycotic and actinomycotic mycetoma, if performed before surgery.

Interestingly, none of the complete responders relapsed during a mean of 73 months follow up. We therefore suggest to take into account not only clinical parameters, but also MRI features before treatment discontinuation as clinical assessment alone was unable to appropriately classify responders, 50% of patients without symptom still having abnormal MRI. Moreover, patients with serum BG or PET/CT major responses also had significantly more chance to achieve a sustained complete response, without relapse. We and others already reported the contribution of serum BG and TEP TDM for follow up of invasive yeast or mold fungal infections [37], [38]. Specific contribution of these tools to customize last generation triazole therapy duration in eumycetoma remains however ill defined and will require prospective studies. Unfortunately, modern triazoles, as MRI or PET/CT are currently too expensive to be largely used in the context of low-income countries. However, prospective validation of the value of negative BD results for eumycetoma long term cure and safe treatment discontinuation would be a major breaktrough in eumycetoma care and would deserve larger diffusion of this cheaper technique.

Overall tolerance of long-term last generation triazoles was good. Long-term voriconazole prescription has been associated with phototoxicity involving acute skin lesions followed by actinic keratosis and ultimately squamous cell cancer [39]. In our study, with cumulative voriconazole therapy of 27.1±30.5 months, no skin lesion was noticed. Black skin phototype, absence of immunosuppression, and shorter mean duration could explain the absence of cutaneous side effects observed during their careful dermatologic monitoring. In addition, fluoride excess and periostitis have been reported in transplant and hematological patients receiving long-term voriconazole therapy, but not with other fluorinated triazole [40]–[43]. In the present study, no case of musculoskeletal pain, PET/CT hypermetabolism, apparent periostitis or exostoses was noticed. Probably due to the efficacy of the new therapeutics used in this study and the resulting improvement of patient's condition, the loss to follow up in our series was only 3/11, contrasting with 50% rate reported in the literature [30].

### Conclusion

Eumycetoma remains one of the most neglected infectious diseases around the world. In high-income countries, imported eumycetoma frequently presents as a severe and unknown disease, which optimal management by trained specialists should now relies on one of the last generation triazoles, posaconazole or voriconazole, that unfortunately aren’t available yet in endemic countries. Beyond essential basic pharmacokinetic patients explanations, optimal use of these drugs should include personalized dosage adapted to therapeutic drug monitoring results and at least a 9-month duration based on clinical, BG, MRI and PET/CT follow up assessment before discontinuation.

## Supporting Information

Checklist S1 STROBE checklist.
(DOC)
